# Histone deacetylase functions and therapeutic implications for adult skeletal muscle metabolism

**DOI:** 10.3389/fmolb.2023.1130183

**Published:** 2023-03-15

**Authors:** Susanna Molinari, Carol Imbriano, Viviana Moresi, Alessandra Renzini, Silvia Belluti, Biliana Lozanoska-Ochser, Giuseppe Gigli, Alessia Cedola

**Affiliations:** ^1^ Department of Life Sciences, University of Modena and Reggio Emilia, Modena, Italy; ^2^ Institute of Nanotechnology, Department of Physics, National Research Council (CNR-NANOTEC), Sapienza University of Rome, Rome, Italy; ^3^ DAHFMO Unit of Histology and Medical Embryology, Sapienza University of Rome, Rome, Italy; ^4^ Department of Medicine and Surgery, LUM University, Casamassima, Italy; ^5^ Institute of Nanotechnology, National Research Council (CNR-NANOTEC), Lecce, Italy

**Keywords:** HDACs, sarcopenia, type 2 diabetes, neurogenic muscle atrophy, sirtuins, glucose uptake, fatty acid oxidation, metabolic flexibility

## Abstract

Skeletal muscle is a highly adaptive organ that sustains continuous metabolic changes in response to different functional demands. Healthy skeletal muscle can adjust fuel utilization to the intensity of muscle activity, the availability of nutrients and the intrinsic characteristics of muscle fibers. This property is defined as metabolic flexibility. Importantly, impaired metabolic flexibility has been associated with, and likely contributes to the onset and progression of numerous pathologies, including sarcopenia and type 2 diabetes. Numerous studies involving genetic and pharmacological manipulations of histone deacetylases (HDACs) *in vitro* and *in vivo* have elucidated their multiple functions in regulating adult skeletal muscle metabolism and adaptation. Here, we briefly review HDAC classification and skeletal muscle metabolism in physiological conditions and upon metabolic stimuli. We then discuss HDAC functions in regulating skeletal muscle metabolism at baseline and following exercise. Finally, we give an overview of the literature regarding the activity of HDACs in skeletal muscle aging and their potential as therapeutic targets for the treatment of insulin resistance.

## 1 Introduction

Histone deacetylases (HDACs) are evolutionarily conserved enzymes that remove acetylated groups from ε-amino lysines of histone proteins. They regulate gene expression both directly, by affecting chromatin accessibility, and indirectly, by interacting with repressor complexes and/or transcription factors (Verdone et al., 2006). Moreover, HDACs are involved in the deacetylation of non-histone proteins that regulate key cellular processes. Finally, HDACs play a critical role in tissue development and homeostasis as effectors in response to physiological and pathological signals.

Genetic and pharmacological manipulations of HDACs *in vitro* and *in vivo* have highlighted their key role in skeletal muscle metabolic maintenance and adaptation. Skeletal muscle is one of the most adaptive organs that undergoes continuous metabolic changes in response to different functional demands. Aberrant expression and activity of HDACs in muscle have been associated with pathological conditions, such as muscular dystrophies. Importantly, numerous clinical trials involving HDAC inhibitors have brought to light the therapeutic potential of HDAC-inhibiting molecules (Marks, 2010; Sandoná et al., 2016).

In this review, after giving a general overview of skeletal muscle metabolism following exercise or nutritional supplementation, we discuss the various functions of HDACs in skeletal muscle, including their fundamental role in skeletal muscle metabolism, adaptation to exercise, and insulin resistance. We provide an overview of the main metabolic pathways regulating skeletal muscle, and we highlight the HDACs that participate in skeletal muscle metabolic processes and remodeling in response to exercise. Finally, we consider the emerging role of HDACs in metabolic disorders, such as insulin resistance, and discuss the therapeutic potential of HDAC inhibitors in metabolic diseases.

## 2 Classification of HDACs

The human HDAC superfamily consists of 18 members grouped into four classes based on their primary homology to yeast HDACs.

Class I consists of HDACs 1, 2, 3, and 8, which show homology to yeast HDAC Rpd3. All of them are ubiquitously expressed, and are predominantly localized within the nucleus. The class I members regulate gene transcription through association with four major corepressor complexes. HDAC1 and HDAC2 activity is mostly exerted through their recruitment into Sin3A, CoREST and NuRD complexes (Yang and Seto, 2008), whereas HDAC3 becomes enzymatically active only when recruited into the SMRT/NCoR repression complex (Oberoi et al., 2011).

Class II, which encompasses HDACs 4, 5, 6, 7, 9, and 10, has a high degree of homology to the yeast HDAC Hda1. These HDACs are expressed in a tissue-specific manner and their activity is regulated mainly through their shuttling between the nucleus and the cytoplasm. Class II is further classified in IIa (HDACs 4, 5, 7, and 9) and IIb (HDACs 6 and 10), with HDAC6 having a tandem deacetylase domain. Most of class IIa HDACs play a role in cell differentiation, consistent with their high expression in skeletal muscle, heart, brain and thymus. Their nucleocytoplasmic trafficking is regulated by the interaction with different binding partners, including MEF2, HIF-1alpha and 14-3-3 proteins. Moreover, the behavior of class IIa HDACs is influenced by post-translational modifications, such as phosphorylation, ubiquitination and sumoylation [reviewed in (Yang and Grégoire, 2005)].

The class III HDACs, also known as sirtuins, is represented by SIRT1 through SIRT7 proteins, encoded by genes homologous to the yeast Sir2 gene. All of the class III members depend on NAD + as cofactor, but while SIRT1-3 and SIRT5 catalyze the deacetylation of histone and non-histone proteins, SIRT4 and SIRT6 catalyze protein ribosylation. The SIRT proteins show different cellular localization, with SIRT1, 6 and 7 localizing within the nucleus, SIRT2 in the cytoplasm and SIRT3-5 in the mitochondria (Michishita et al., 2005).

Currently, class IV includes only the HDAC11 member, which shares partial homology with class I and II HDACs. HDAC11 is highly expressed in the brain, heart, skeletal muscle, and kidney, and is localized in the nucleus and muscle mitochondria. Unlike the other HDACs, HDAC11 is more efficient at removing long-acyl than acetyl groups, thereby acting as a long-chain fatty acid deacylase, and thus as a sensor for fatty acid metabolism inside the cell (Núñez-Álvarez and Suelves, 2022).

## 3 Skeletal muscle metabolism

Healthy skeletal muscle can adjust fuel utilization to the intensity of muscle activity, the availability of nutrients and the intrinsic characteristics of muscle fibers. This property, defined as “metabolic flexibility”, guarantees the production of adenosine triphosphate (ATP) for direct use for muscle contraction with maximum yield in a rather wide range of conditions (Goodpaster and Sparks, 2017; Bouchè et al., 2018). ATP is also necessary for ionic transport, especially for calcium uptake by the sarcoplasmic reticulum (SR), and is mainly generated through glucose oxidation by glycolysis and beta-oxidation of fatty acids. The choice between these two metabolic pathways is controlled by hormones, and by nutrients through the glucose-fatty acid cycle, which was first described by Sir Philip John Randle (the Randle cycle). Randle et al. proposed (Randle et al., 1963) that this dynamic adaptation to nutrient availability is based on the interaction between adipose and muscle tissues. According to this model, hormones that control adipose tissue lipolysis regulate the concentration of circulating fatty acids, which in turn, controls fuel selection in muscle. Moreover, fuel catabolism is governed by the circadian clock in skeletal muscle; however, the underlying molecular mechanisms are still largely unknown (Harfmann et al., 2015; Mayeuf-Louchart et al., 2015).

**FIGURE 1:**
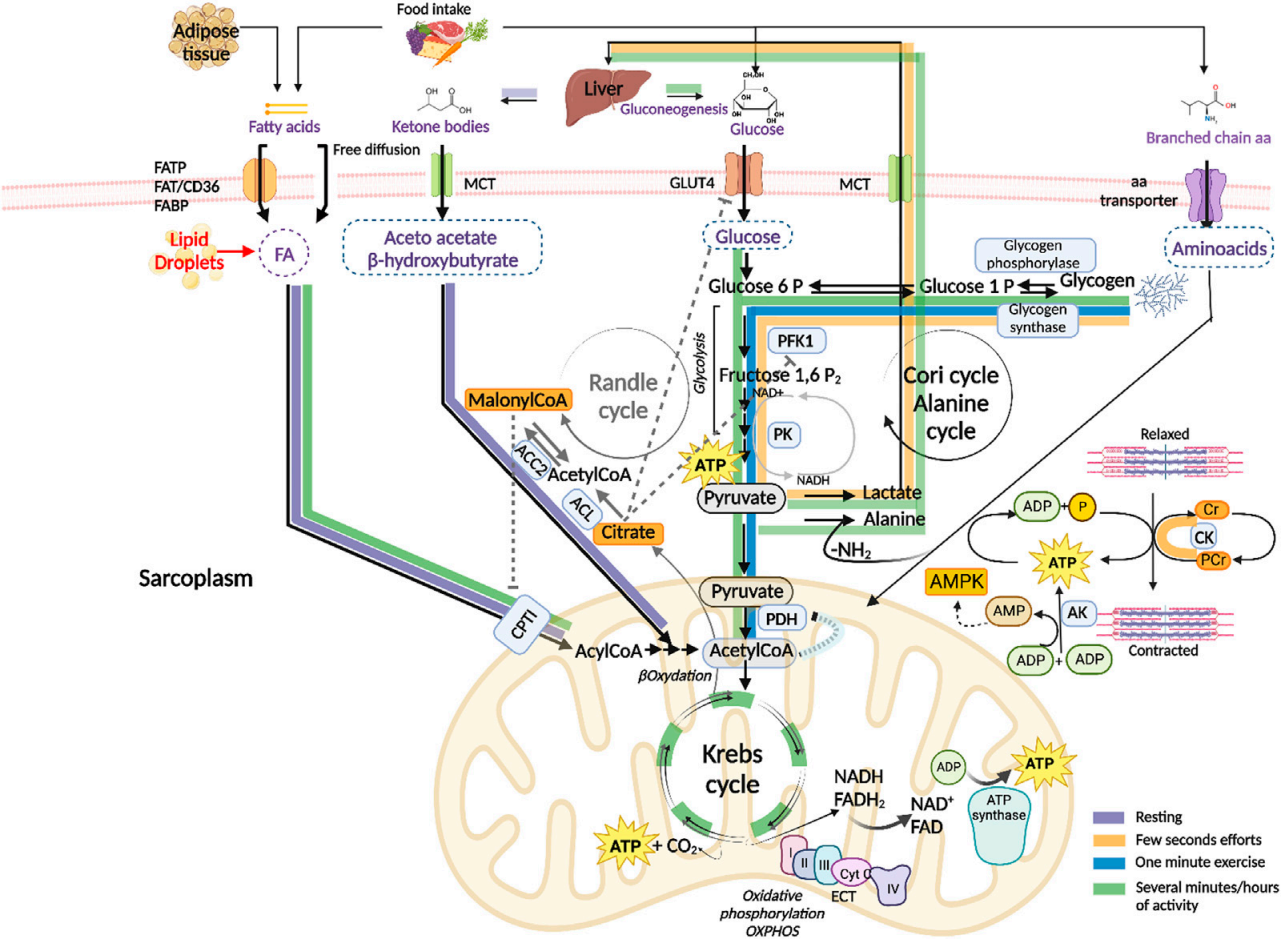
Schematic presentation of the energy substrates used by skeletal muscle to produce adenosine triphosphate (ATP) under different conditions. ATP hydrolysis to Adenosine diphosphate (ADP) and phosphate (P), catalyzed by myosin ATPase, provides the energy necessary for skeletal muscle contraction Purple arrows: the main energy sources used by resting muscle are fatty acids from adipose tissue and ketone bodies from liver. After passing the sarcolemma, fatty acids enter the mitochondria where they are degraded to acetyl CoA (βoxidation) and completely oxidized to carbon dioxide in the Krebs cycle which generates ATP and the reduced forms of the nicotinamide adenine dinucleotide (NAD) and flavin adenine dinucleotide (FAD) cofactors. NADH and FADH_2_ oxidation by the electron transport chain (ETC.) is coupled to ATP synthesis (oxidative phosphorylation, OXPHOS). Glucose is used to replenish the glycogen stores by glycogen synthase. Fatty acid–binding protein (FABP), fatty acids transport proteins (FATPs), fatty acids translocase (FAT/CD36), monocarboxylate transporters (MCTs), carnitine palmitoyltransferase I (CPTI). Orange arrows: in the very early seconds of activity, degradation of phosphocreatine (PCr) by creatine kinase (CK) releases free Cr and P, which is transferred to ADP to regenerate ATP. The adenylate kinase (AK) enzyme catalyzes the formation of ATP and Adenosine monophosphate (AMP) from two ADP molecules. AMP activates the AMP kinase (AMPK), a key sensor of the energy level the cell. ATP is also generated by anaerobic glycolysis, where glucose-6-phosphate derived from muscle glycogen (by means of glycogen phosphorylase) is catabolized to pyruvate by glycolytic enzymes including phosphofructokinase 1 (PFK1) and pyruvate kinase (PK), which is reduced to lactate. Lactate is released into the circulation and taken up by the liver for the synthesis of glucose through gluconeogenesis (Cori cycle). Blue arrows: when high power exercise lasts beyond one minute, ATP is mainly generated by oxidative metabolism of glycogen-derived glucose. Pyruvate is transported to the mitochondrion and transformed into acetyl CoA by the pyruvate dehydrogenase (PDH) complex and enters the Krebs cycle. Green arrows: in case of prolonged exercise, ATP is generated by the oxidative metabolism of both carbohydrates and fatty acids from endogenous and exogenous stores. Branched-chain amino acids are transformed into ketoacids by a specific transaminase. The ammonia group is transferred to pyruvate generating alanine which is transported to the liver and used as a gluconeogenetic precursor (alanine cycle). Randle cycle: allosteric mechanisms underlying fuel selection for ATP supply by skeletal muscle involve acetyl CoA, Citrate and malonyl CoA, they are indicated by dashed grey inhibitory lines and grey arrows. ATP citrate lyase (ACL), acetylCoA carboxylase 2 (ACC2). Created with BioRender.com.

The main energy source in resting muscle is represented by fatty acids and ketone bodies, produced by adipose tissue and liver, respectively. Fatty acids enter muscle fibers by passive diffusion or by protein-mediated transport, mainly involving the plasma membrane fatty acid-binding protein (FABP), fatty acid-transport proteins (FATP1 and FATP6) and fatty acid translocase (FAT/CD36). Upon entry into the cells, they are translocated into mitochondria by carnitine palmitoyltransferase I (CPTI). They undergo oxidation and degradation to acetyl coenzyme A (acetyl CoA), which is subsequently oxidated to carbon dioxide in the Krebs cycle which generates ATP and the reduced forms of the nicotinamide adenine dinucleotide (NAD) and flavin adenine dinucleotide (FAD) cofactors that carry high energy electrons to the electron transport chain (ETC.) where they are used to synthesize ATP molecules (oxidative phosphorylation, OXPHOS). In these conditions, glucose and amino acids are mainly used to replenish the glycogen stores (Figure 1, purple arrows).

### 3.1 Fuel utilization during exercise

Exercise is a physiological condition that requires metabolic flexibility to adapt to fuel availability and meet higher energy demands. At the highest power output, ATP consumption increases up to a hundred times compared to the resting state. The type of fuel used during exercise depends on the type, intensity and duration of the exercise, as well as on muscle fiber composition, in addition to individual factors, such as sex, age, and nutritional and environmental conditions [reviewed in (Hargreaves and Spriet, 2020)]. Generally, the transition from rest to exercise causes skeletal muscle to switch fuel utilization in favor of carbohydrates and to a lesser extent fatty acids, since carbohydrates result in higher ATP production (Goodpaster and Sparks, 2017). The switch is guaranteed by short term allosteric and covalent regulatory mechanisms as well as by long term control of the expression of key enzymes, which are collectively indicated with the name of the Randle cycle. The main allosteric mechanisms underlying the inhibition of glucose catabolism by fatty acids is based on the accumulation of acetyl CoA and citrate which cause respectively an inhibition of the pyruvate dehydrogenase (PDH) complex, of GLUT4-mediated glucose uptake and phosphofructokinase 1 (PFK1) enzyme (Figure 1 dashed grey lines). On the other hand, glucose metabolism leads to an accumulation of malonyl CoA thanks to the activities of the ATP citrate lyase (ACL) and the acetyl CoA carboxylase 2 (ACC2) enzymes. Malonyl CoA in turn inhibits the entry of fatty acids into the mitochondrion by CPTI, slowing down their oxidation (Hue and Taegtmeyer, 2009) (Figure 1, grey arrows).

In the very early seconds of high-power activity, skeletal muscle uses phosphocreatine (PCr), a phosphorylated form of creatine (Cr) that serves as a rapidly mobilizable reserve of high-energy phosphates to regenerate ATP by means of the creatine kinase (CK) enzyme. During muscle contraction ATP is hydrolyzed to ADP; the consequent increase in ADP concentration stimulates cellular respiration and provides an additional source of ATP: the adenylate kinase (AK) enzyme catalyzes the reversible formation of ATP and adenosine monophosphate (AMP) from two adenosine diphosphate (ADP) molecules. AMP is an allosteric regulator of several metabolic enzymes, including glycogen phosphorylase and PFK1. AMP activates the AMP kinase (AMPK), a key sensor of the energy level the cell which stimulates catabolic processes while inhibiting anabolic pathways. Indeed, AMPK promotes glucose transport, fatty acid oxidation, and mitochondriogenesis while inhibiting glycogen and protein synthesis [reviewed in (Kjøbsted et al., 2018)]. ATP is also generated by anaerobic glycolysis, where glucose-6-phosphate derived from muscle glycogen is catabolized to pyruvate which is reduced to lactate. Lactate is released into the circulation and taken up by the liver for the synthesis of glucose through gluconeogenesis (Cori cycle) (Figure 1, orange arrows).

When high-intensity exercise is prolonged beyond one minute, or during moderate activity, blood flux increases and glycogen-derived glucose is the major energy source that generates pyruvate. The latter is transported to the mitochondrion and transformed into acetyl CoA by the PDH complex, which feeds the Krebs cycle and oxidative phosphorylation (Figure 1, blue arrows). In conditions of long-lasting and intense muscular activity, the energy demand becomes very high, and ATP is obtained through oxidative metabolism of both carbohydrates (from muscular and hepatic glycogenolysis and liver gluconeogenesis) and fatty acids (Figure 1, green arrows). Free fatty acids mostly derive from triglycerides released from adipose cells, they are also released from intramuscular triglycerides primarily during prolonged muscle contraction. The contribution of branched-chain amino acids (BCAAs) (valine, leucine and isoleucine) to ATP regeneration is generally low but it increases in contracting muscle when the amount of carbohydrates is very low. Skeletal muscle contains a specific transaminase which transforms them into ketoacids which can then be oxidized into the mitochondria in the Krebs cycle. The ammonia derived from BCAAs degradation is transferred to pyruvate generating alanine which is transported to the liver where its carbon skeleton is used as a gluconeogenetic precursor (alanine cycle).

Intense muscle activity takes place under the control of adrenaline that promotes muscle glycogenolysis and inhibits liver glycolysis, thus stimulating a hyperglycemic response. Anaerobic glycolysis assumes essential significance for the availability of ATP in strenuous physical activity, when the demand for ATP exceeds the muscle’s oxidative capacity and the availability of oxygen. Glucose is synthesized by liver through the Cori cycle, which, together with the alanine cycle, integrates the metabolic functions of skeletal muscle and liver.

### 3.2 The effects of endurance exercise on skeletal muscle metabolism

Skeletal muscle is a metabolically flexible and plastic organ, and therefore highly adaptable to physical exercise. Skeletal myofibers can be classified as type I slow-twitch or type II fast-twitch fibers based on the expression of myosin heavy chain (MHC) isoforms. Slow myofibers generally express type I myosin, whereas fast myofibers can be further classified into three subtypes, depending on the expression of myosin IIa, IIx, or IIb. Human skeletal muscle lacks the fastest type IIb fibers (Schiaffino, 2010; Schiaffino and Reggiani, 2011). Skeletal myofibers also differ in their metabolic properties: type I and type IIa myofibers are oxidative, a property that depends on a reserve of oxygen bound to myoglobin and a high content of mitochondria. Fast type IIx and IIb fibers use glycolysis as their main pathway to obtain energy. Myofiber subtypes arise during development, but their phenotype and size can change in response to metabolic conditions and exercise, a property that is defined as muscle plasticity.

In this regard, aerobic exercise activates the AMPK in a chronic manner, leading to the remodeling of fibers from glycolytic to oxidative. This transition involves specific transcriptional events, including the activation of the Ca2+/calmodulin-dependent protein kinases (CAMKII and CAMKIV) and the consequent activation of the transcription factor NFAT. Similarly, physical exercise determines the activation of the transcription factor MEF2, by promoting the class IIa HDAC nuclear extrusion, and of other important regulators of metabolic gene expression, such as peroxisome proliferator-activated receptors (PPARs) and peroxisome proliferator-activated receptor-gamma coactivator (PGC)-1alpha. As a result, exercise enhances the expression of a series of genes important for lipid oxidation, such as FAT, fatty acid synthase (FASN), CPTI. Furthermore, AMPK-dependent activation of PGC-1alpha leads to mitochondriogenesis (Lin et al., 2002; Wu et al., 2002; Ljubicic et al., 2011).

In conclusion, endurance exercise is accompanied by several physiological adaptations that improve muscle function and tissue performance. Trained muscle shows a remodeling towards a more oxidative phenotype.

### 3.3 Skeletal muscle metabolism and nutrition

The alternation between feeding and fasting phases requires a high skeletal muscle metabolic flexibility. The transition from fasting to a fed state is accompanied by a change in fuel utilization from oxidative fatty acid metabolism to glucose oxidation. Insulin secretion that follows a carbohydrate-rich food intake provides an integrated set of signals that balance the availability of nutrients and energy demands. Skeletal muscle represents one of the most effective targets of insulin, where it stimulates GLUT4-mediated glucose transport and glycogen synthesis.

By contrast, during the fasting phase, the preferred fuel of skeletal muscle is free fatty acids released by the adipose tissue and ketone bodies synthesized by the liver. Fatty acids and ketone bodies inhibit the utilization of carbohydrates in order to save glucose for the brain (Randle cycle). Moreover, fasting activates autophagy in skeletal muscle, which enables alternative cellular energy stores to produce nutrients in times of need. In this regard, autophagy is involved in glycogen degradation, glucose utilization, lipid metabolism, and mitochondrial function, overall influencing muscle metabolism and exercise-induced muscle adaptations (Sebastián and Zorzano, 2020).

### 3.4 Metabolic alterations of skeletal muscle and disease

In addition to its role in body movement, skeletal muscle metabolism and its metabolic flexibility play a key role in whole-body metabolic homeostasis. It is believed that excessive nutrient consumption is a major cause of muscle metabolic inflexibility, which leads to the accumulation of intermediates like diacylglycerol (DAG) and ceramide, a sphingolipid derivative of palmitate, that interfere with insulin signaling, resulting in insulin resistance (Muoio and Newgard, 2008; Samuel and Shulman, 2012). Loss of metabolic flexibility is frequently accompanied by altered mitochondrial metabolism, which also contributes to insulin resistance (Kelley and Mandarino, 2000; Muoio, 2014). Given that skeletal muscle is responsible for about 60%–80% of the increase in glucose metabolism in response to insulin, it is unsurprising that a decrease of insulin sensitivity in this organ is a major cause of type 2 diabetes (T2D) (Wolfe, 2006; DeFronzo and Tripathy, 2009).

## 4 HDACs in skeletal muscle metabolism

HDACs regulate muscle metabolism, targeting many metabolic enzymes (Guan and Xiong, 2011) and more than 20% of mitochondria proteins (Choudhary et al., 2009). Most HDAC functions in skeletal muscles have been identified by genetic tools, *in vitro* and *in vivo*. Not all members of the HDAC superfamily play a role or have been studied in skeletal muscle metabolism; below we review only the ones whose functions have been elucidated.

### 4.1 Class I HDACs

HDAC1 and HDAC2 play redundant roles in skeletal muscle metabolism by mediating autophagosome formation and ensuring a proper autophagic flux. Deletion of both genes induces perinatal lethality in a subset of skeletal muscle-specific double-knockout (KO) mice, associated with mitochondrial abnormalities and sarcomere degeneration (Moresi et al., 2012). Mutant mice surviving through the first days of life develop a progressive myopathy characterized by higher energy expenditure, which is fully rescued by feeding the mice a high-fat diet (HFD), likely prolonging caloric availability and diminishing the requirement for autophagy in skeletal muscle. Importantly, the deletion of HDAC1 and HDAC2 in skeletal muscle induces only a modest change in gene expression, mainly related to metabolism, suggesting that these epigenetic factors do not function as global repressors of gene transcription (Moresi et al., 2012).

HDAC3 regulates the metabolism of different tissues, including the liver and cardiac muscle (Knutson et al., 2008; Montgomery et al., 2008). Likewise, in postnatal skeletal muscle, HDAC3 is a master transcriptional regulator that couples the circadian clock with muscle metabolism (Hong et al., 2017). HDAC3 binds and transcriptionally regulates key genes involved in amino acid catabolism and glucose uptake, in a circadian pattern. Skeletal muscle-specific deletion of HDAC3 induces insulin resistance and impaired glucose uptake following exercise, and enhances fatty acid oxidation. Skeletal muscle-specific HDAC3 KO mice develop a fuel preference toward lipids, rather than glucose, increasing amino acid catabolism, which leads to muscle mass loss with age. Such functions are dependent on HDAC3 deacetylase activity (Song et al., 2019).

The importance of HDAC3 in skeletal muscle metabolism has been further clarified by studies in which its expression is upregulated in skeletal muscle. Upon free fatty acid or high fat and fructose diet, HDAC3 expression is increased in skeletal muscle and mediates a fatty acid-induced impairment of mitochondrial oxidation, resulting in increased generation of reactive oxidative species and accumulation of intracellular triglycerides (Lee et al., 2020). As expected, HDAC3 knockdown prevents free fatty acid-induced insulin resistance in myotubes, and diet-induced inflammation (Lee et al., 2020). By contrast, HDAC3 KO mice that lack HDAC3 in postnatal skeletal muscle are prone to death on a HFD, likely due to a deficiency in mitochondriogenesis (Sun et al., 2011). The different experimental models - HDAC3 genetic deletion driven by muscle creatine kinase in one, and siRNA *in vitro* in the other - may account for these contradictory conclusions. Nevertheless, all these studies prove the importance of balanced HDAC3 expression levels in skeletal muscle metabolism.

### 4.2 Class II HDACs

An elegant genetic study defined the redundant role of class IIa HDACs in repressing the MEF2-dependent activation of the oxidative-fiber phenotype (Potthoff et al., 2007). Selective proteasome-degradation (Potthoff et al., 2007) and intracellular localization (Cohen et al., 2015) of class IIa HDACs finely tune the MEF2-dependent, PGC-1alpha-mediated oxidative metabolic gene program. Moreover, HDAC5 inhibits the expression of Glut4 and Baf60c, thereby influencing muscle glucose uptake (Raichur et al., 2012; Meng et al., 2017). Forced expression of the catalytic active-site mutants of HDAC4 and HDAC5, which are no longer able to bind the SMRT/NCoR repression complex, induce MEF2 activity and oxidative muscle metabolism (Gaur et al., 2016).

HDAC6 is a crucial regulator of autophagosome-lysosome fusion (Lee et al., 2010) and mediates mitochondrial fusion (Lee et al., 2014). In physiological conditions, HDAC6 deletion causes increased production of mitochondrial reactive oxygen species (ROS) in mice (Lee et al., 2014). Upon fasting, HDAC6 deletion results in extensive mitochondrial degeneration due to a defect in mitochondrial fusion (Lee et al., 2014). In diabetic mice, HDAC6 expression in skeletal muscle is upregulated, and impairs insulin signaling by downregulating Insulin Receptor Substrate 1 (IRS1) levels (Kumar and Datta, 2022). This study provided further evidence that HDAC6 plays an important role in muscle metabolism.

### 4.3 Class III HDACs

Each member of the class III HDACs, or sirtuins, plays distinct roles in body metabolism, despite having a conserved catalytic domain. All members have been somehow implicated in cellular responses to energy demand: while the nuclear SIRT1, SIRT6, and SIRT7 act as transcription factors or cofactors, the mitochondrial SIRT3, SIRT4, and SIRT5 modulate the activity of mitochondrial enzymes. Here we give a brief overview of the members directly implicated in skeletal muscle metabolism.

Mice carrying null alleles for SIRT1 clarified its importance in regulating energy metabolism and adequate responses to caloric restriction (Boily et al., 2008). Being NAD + -dependent deacetylase, SIRT1, together with AMPK (Price et al., 2012), represents a sensing node for skeletal muscle that adapts its responses to external and cellular substrate availability, regulating the activity of numerous proteins involved in muscle metabolism and mitochondriogenesis, including PGC-1alpha (Nemoto et al., 2005). The SIRT1-PGC-1alpha axis is indeed one of the main regulators of energy expenditure, mainly through the expression and translocation of the insulin-sensitive glucose transporter Glut4 (Michael et al., 2001), which is considered the limiting step for muscle glucose uptake, and of numerous mitochondrial genes. Low glucose concentration triggers SIRT1 activation, which in turn promotes fatty acid *ß*-oxidation, by deacetylating FOXO1 and PGC-1alpha in skeletal muscle (Gerhart-Hines et al., 2007). Conversely, SIRT1 expression is decreased in insulin-resistant obese mice and inversely correlates with the expression of protein tyrosine phosphatase 1b, a major inhibitor of the insulin receptor, thereby influencing the insulin response (Sun et al., 2007).

By contrast, SIRT2 negatively regulates insulin resistance in skeletal muscle cells (Arora and Dey, 2014). SIRT2 KO mice display alterations in blood glucose levels (Lee et al., 2022) and, when subjected to HFD, exacerbate insulin resistance (Lantier et al., 2018). However, the mechanisms underlying SIRT2-mediated regulation of muscle metabolism are still unclear.

SIRT3 is predominantly expressed in slow-oxidative myofibers (Palacios et al., 2009). SIRT3 KO mice display insulin resistance caused by increased oxidative stress, activation of stress kinase JNK, and decreased respiration (Jing et al., 2011). Moreover, SIRT3 KO mice possess muscle-impaired ability to adjust to fuel oxidation (Muoio and Neufer, 2012). By targeting and thereby affecting the activity of PDH, SIRT3 modulates muscle metabolism. Indeed, SIRT3 KO mice prefer fatty acid oxidation, even in the presence of glucose (Jing et al., 2013; Lantier et al., 2015). Like SIRT1, SIRT3 expression is finely regulated by metabolic cues in skeletal muscle - upregulated by caloric restriction, fasting, or exercise training, and downregulated by HFD. Depending on the metabolic demand, SIRT3 dynamically mediates insulin sensitivity and energy response (Jing et al., 2011; Lantier et al., 2015).

SIRT4 negatively regulates mitochondriogenesis and fatty acid oxidation in muscles, likely by repressing SIRT1 expression and thus targeting AMPK activation (Nasrin et al., 2010).

Mice lacking SIRT6 die before 1 month of age due to hypoglycemia (Mostoslavsky et al., 2006). SIRT6 maintains efficient glucose flux into the TCA cycle by acting as a HIF-1alpha corepressor and modulating the histone deacetylation at the HIF-1alpha target gene promoters. SIRT6 deficiency causes a cell-autonomous upregulation of glucose uptake also in skeletal muscle, leading to a metabolic switch toward glycolysis, thus circumventing mitochondrial respiration (Zhong et al., 2010). SIRT6 skeletal muscle-specific KO mice further confirmed the physiological importance of this enzyme as a regulator of muscle mitochondrial function, promoting AMPK-dependent fatty acid uptake and oxidation (Cui et al., 2017).

Thus, while SIRT1 and SIRT6 positively regulate glucose uptake and fatty acid oxidation in skeletal muscle, SIRT1 activity is inhibited by SIRT4 and balanced by SIRT3, which inhibits fatty acid oxidation and affects ROS production, thereby influencing insulin resistance.

### 4.4 Class VI HDACs

Despite being dispensable for muscle development or maintenance, HDAC11 regulates mitochondrial fatty acid oxidation by inhibiting the AMPK-acetyl-CoA carboxylase pathway (Hurtado et al., 2021). HDAC11 deletion induces a myofiber shift toward a slower-oxidative, fatigue-resistant phenotype (Hurtado et al., 2021). Moreover, HDAC11 KO mice are resistant to HFD-induced obesity and metabolic syndrome, presenting higher energy expenditure (Bhaskara, 2018).

**FIGURE 2:**
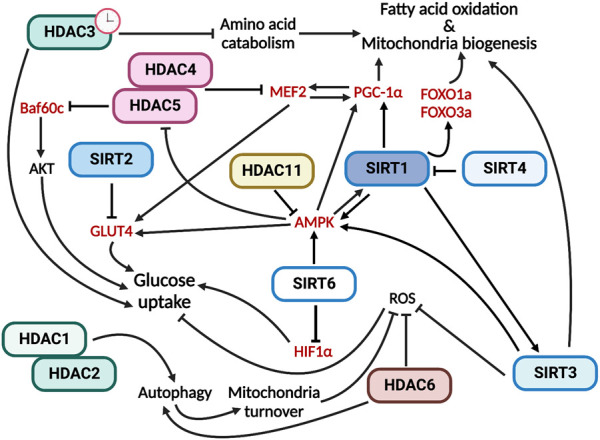
HDACs have multiple and specific functions in regulating skeletal muscle metabolism. In red are indicated the HDAC direct targets.

In conclusion, all members of the HDAC family are involved in the regulation of skeletal muscle metabolism by performing specific functions (Figure 2).

## 5 HDACs in skeletal muscle during exercise

Exercise triggers various adaptive responses in skeletal muscle allowing it to meet different energy demands determined primarily by exercise intensity and duration. Exercise enhances substrate uptake and produces ATP through mitochondrial oxidation or anaerobic pathways in the case of strength exercise. During energy deficits, skeletal muscle experiences a severe decrease in glucose availability that needs to be compensated by an increase in mitochondrial fatty acid oxidation to preserve glucose for glucose-dependent tissues.

The extensive metabolic and molecular remodeling of skeletal muscle by exercise depends on transient changes in gene expression that promote the mitochondrial use of lipid substrates as a source of energy. Acute exercise elicits a rapid and transient transcriptional activation of specific genes, the expression of which peaks 3–12 h after cessation of the exercise and returns to basal level within 24 h (Egan and Zierath, 2013). McGee and coworkers demonstrated that exercise induces chromatin remodeling associated with enhanced transcription, consistently with increased H3K36 acetylation (McGee et al., 2009). Long-term adaptation to training induces changes mainly in protein content or enzyme function rather than in transcript expression, due to a gradual protein accumulation in response to a repeated increase in mRNA levels. Moreover, skeletal muscle response to physical exercise depends on muscle fiber types that are characterized by different metabolic profiles.

The expression levels of HDACs and the effects of their deletion *in vivo* and *in vitro* demonstrate their different roles in the metabolic profile and exercise response of skeletal muscle (Table 1).

**TABLE 1:** Role of HDACs in skeletal muscle following exercise identified by different mouse models. SkM, skeletal muscle, KO, knock out, KI, knock in. All the described genetically engineered mouse models were generated by germline manipulation.

Enzyme	*In vivo* mouse model	Effect of KO or KI on		References
		Exercise performance	Metabolism during exercise	
HDAC3	*Hdac3* SkM-KO NS-DADm KI	**↓**muscle force	**↓**glucose uptake	Hong et al. (2017); Song et al. (2019)
		**↑**exercise endurance fatigue resistance	**↑**lipid oxidation	
HDAC4 HDAC5	*Hdac4* SkM-KO *Hdac5* KO	**↑** exercise performance	**↑** fatty acid oxidation	Potthoff et al. (2007); Gaur et al. (2016)
		**↑**whole-body energy expenditure	**↓** plasma lipids and glucose	
SIRT 1	*Sirt1* SkM-KO	**=** muscle endurance	**=** oxidative capacity	Philp et al. (2011)
SIRT3	*Sirt3* KO	**↓** exercise endurance	**↓** mitochondrial respiration and ATP production	Vassilopoulos et al. (2014)
SIRT4	*Sirt4* KO	**↓** exercise performance	**↑** lipid oxidation	Laurent et al. (2013)
SIRT6	*Sirt6* SkM-KO	**↓** exercise performance	**↓** glucose and lipid uptake	Cui et al. (2017)
			**↓** fatty acid oxidation	
			**↓** mitochondrial oxidative phosphorylation	
HDAC11	*Hdac11* KO	**↑**exercise endurance fatigue resistance	**↑**fatty acid oxidation	Hurtado et al. (2021)

### 5.1 Class I HDACs

Class I HDAC1, 2, and 3 are expressed at similar level in all muscle groups (Potthoff et al., 2007). While the possible and specific functions of HDAC1 and HDAC2 in response to exercise remain unexplored, the role of HDAC3 in skeletal muscles has been well characterized. HDAC3 depletion results in lower glucose uptake during muscle contraction and greater lipid oxidation, which eventually reduces muscle force but enhances exercise endurance and fatigue resistance (Hong et al., 2017; Gong et al., 2018). Specifically, enhanced fatty acid oxidation is associated with upregulated expression of metabolic genes involved in the catabolism of branched-chain amino acids (BCAAs). Despite robust metabolic alterations, neither change in muscle fiber-type composition nor mitochondrial protein content is observed. Interestingly, the mutation of the NCoR/SMRT corepressors in a knock-in mouse model (NS-DADm) that abolishes the enzymatic activity of HDAC3 without affecting its protein expression, reproduces the metabolic phenotype of the HDAC3 KO mouse. The NS-DADm is indeed characterized by lower force generation, enhanced fatigue resistance and fatty acid oxidation rate, lower glucose uptake during exercise, and upregulated expression of metabolic genes involved in BCAAs catabolism (Song et al., 2019). These results clearly demonstrate that HDAC3 enzymatic activity *per se* regulates muscle fuel metabolism (Gong et al., 2018).

### 5.2 Class II HDACs

Class IIa HDAC4, 5 and 7, are preferentially expressed in fast muscle fibers (Potthoff et al., 2007). Compared to class I, class IIa HDACs have lower deacetylase activity against acetyl-lysine substrates, hence they are complexed with SMRT/N-Cor and HDAC3 to exert their repressive activity (Kao et al., 2000; Fischle et al., 2002) The total activity of class IIa HDACs is not altered during exercise. Despite this, Potthoff et al. showed that ubiquitin-mediated proteasomal degradation of class IIa HDACs contributes to skeletal muscle adaptation in response to exercise training (Potthoff et al., 2007). In human skeletal muscle, ubiquitin-mediated proteasomal degradation of HDAC5 occurs immediately following exercise, affecting chromatin remodeling in the post-exercise period (McGee et al., 2009). Moreover, during exercise, the release of Ca2+ from the sarcoplasmic reticulum activates calcium/calmodulin-dependent protein kinase (CaMK), which in turn phosphorylates and induces nuclear export of HDAC4 and HDAC5, thus disrupting the repressive complex (McGee et al., 2009). The key role of HDAC5 is further demonstrated by gain-of-function experiments, in which HDAC5 over-expression inhibits skeletal muscle adaptation to exercise training (Potthoff et al., 2007).

Various studies have highlighted that HDACs 4, 5, 7, and 9 repress gene expression by interacting with the MEF2 transcription factors (McKinsey et al., 2001; Potthoff et al., 2007). The class IIa HDACs/MEF2 axis is pivotal in adaptations to exercise. McGee and coworkers showed that exercise-induced nuclear export of HDAC4 and HDAC5 reduces their association with MEF2, leading to an increase in its ability to bind and transactivate MEF2-dependent genes (McGee and Hargreaves, 2004; McGee et al., 2008; McGee et al., 2009). Genetic and pharmacological disruption of the class IIa HDAC corepressor complex increases the expression of exercise-responsive genes, enhances exercise performance, and induces metabolic adaptations by increasing whole-body energy expenditure and fatty acid oxidation, and decreasing blood lipids and glucose (Gaur et al., 2016). A single bout of exercise in human skeletal muscle leads to HDAC4/5 nuclear export and activation of MEF2A, which in turn triggers the expression of Glut4 (McGee et al., 2008), thus mediating glucose uptake. Similarly, exercise-induced Glut4 transcription is triggered by AMPKalpha2-dependent inactivation of HDAC4/5 in mouse muscle (Niu et al., 2017). Another target of the HDAC5/MEF2A axis during exercise is the Cpt1b (muscle carnitine palmitoyltransferase-1b) gene, which encodes a key enzyme in the regulation of skeletal muscle mitochondrial *ß*-oxidation of long-chain fatty acids. Its expression increases in the quadriceps muscle of mice after a 6-week aerobic exercise intervention, while the binding of HDAC5 to the Cpt1b promoter on the MEF2-binding site decreases following exercise training, in parallel with MEF2A-induced Cpt1b transcription (Yuan et al., 2014).

### 5.3 Class III HDACs

Sirtuins are very sensitive to metabolic alterations as a consequence of their functional dependency on NAD+ and play a key role in metabolic adaptation. SIRT1 and SIRT3 are the most studied sirtuins in muscle metabolism. Regardless of age, regular exercise increases the activity and expression of SIRT1 and 3, thus improving the efficiency of oxidative metabolism and mitochondrial biogenesis and function (Boily et al., 2008; Lee et al., 2014; Kumar and Datta, 2022). Despite this general consideration, the effect of exercise on SIRT1 and 3 in human skeletal muscle depends on the type of exercise (Vargas-Ortiz et al., 2019).

SIRT1 is one of the master regulators of exercise-induced beneficial effects (Bayod et al., 2012). The function of the nuclear SIRT1 protein in the control of glucose homeostasis, lipid metabolism, and oxidative capacity relies on the modulation of the acetylation status of the metabolic coregulator PGC-1alpha that enhances its activity (Nemoto et al., 2005; Suwa et al., 2008). Indeed, SIRT1 regulates AMPK and in turn PGC-1alpha activity, which controls skeletal muscle mitochondrial function increasing energy expenditure and exercise performance (Lira et al., 2010). The physiological activation of AMPK in skeletal muscle during exercise significantly increases intracellular NAD+, confirming the interdependence of AMPK and SIRT1 during metabolic adaptation to exercise (Cantó et al., 2010). Together with mitochondriogenesis, SIRT1 transcript, protein, and activity increase in skeletal muscle during endurance exercise training, (Koltai et al., 2010). Similarly, SIRT1 is involved in skeletal muscle metabolic and performance adaptations following high-intensity interval training (Little et al., 2010). However, discordant data were obtained from muscle-specific SIRT1 KO mice, in which neither deacetylation of PGC-1alpha nor skeletal muscle endurance and oxidative capacity are affected. These data suggest that GCN5 acetyltransferase, rather than SIRT1, is the key regulator of PGC-1alpha activity following exercise (Philp et al., 2011).

SIRT3 is more expressed in type I muscle fibers. In human muscles, SIRT3 expression does not change following acute and short-duration endurance training (>6 weeks), while it increases upon long-duration endurance training (>8 weeks) [reviewed in (Zhou et al., 2022)]. Like in humans, in rats, levels change based on different training and muscle type. A single bout of treadmill running does not affect SIRT3 expression, while endurance treadmill-running training increases SIRT3 protein in soleus and plantaris muscles. Likewise, voluntary wheel-running training increases SIRT3 protein in plantaris and triceps muscles (Hokari et al., 2010) These results suggest that SIRT3 abundance is associated with muscle aerobic capacity. Also in mouse muscles, SIRT3 expression dynamically changes following voluntary exercise, triggering downstream molecular responses (Palacios et al., 2009). Data generated from SIRT3 KO mice have highlighted that SIRT3 modulates muscle energy homeostasis following exercise through AMPK and PGC-1alpha, similarly to SIRT1. In addition, exercise may alter SIRT3-mediated deacetylation of the F1 portion of the ATP synthase complex that, once deacetylated, increases mitochondrial respiration and ATP production (Vassilopoulos et al., 2014).

In SIRT4 KO mice there is a metabolic shift toward lipid utilization, with increased malonyl CoA decarboxylase and consequently reduced malonyl CoA, which regulates the switch between fatty acid synthesis and oxidation. As a result, SIRT4-depleted mice demonstrate higher exercise capacity, and increased lipid oxidation (Laurent et al., 2013).

The contribution of SIRT6 to exercise performance is highlighted by muscle-specific KO mice, which run for shorter periods of time and distance compared with control mice, demonstrating decreased endurance exercise capacity (Cui et al., 2017).

### 5.4 Class IV HDACs

Studies on the HDAC11 KO mouse model demonstrate increased fatty acid oxidation capacity in skeletal muscle, which usually occurs during prolonged exercise (Hurtado et al., 2021). Consistently, HDAC11-deficient mice have shown higher muscle endurance when subjected to increased exercise load on a treadmill. In addition to increased fatigue resistance, the absence of HDAC11 increases skeletal muscle strength (Hurtado et al., 2021).

## 6 HDACs as targets for the treatment of insulin resistance

In addition to mediating the crosstalk between muscle and other tissues and organs, HDACs regulate metabolism in different tissues, such as the liver, skeletal muscle, white and brown adipose tissues, (Renzini et al., 2022). Due to their prominent role, pharmacological modulation of HDAC activity represents a potential therapeutic approach for the treatment of metabolic diseases, such as insulin resistance, obesity, and type 2 diabetes (Ye, 2013; Dewanjee et al., 2021).

The pan-HDACi sodium butyrate improves HFD-induced obesity and insulin resistance in mice, by activating PGC-1alpha and AMPK (Gao et al., 2009), and promoting the transcription of the IRS1 gene (Chriett et al., 2017), thereby increasing glucose uptake, mitochondriogenesis and energy expenditure in skeletal muscle. Interestingly, IRS1 has also been identified as a target of HDAC6, and its modulation by the pan-HDACi SAHA rescues diabetic (db/db) or HFD-induced obesity in mice (Kumar and Datta, 2022).

Similarly, the class I HDACi helminthosporium carbonum (HC) toxin activates the IRS1-Akt and AMPK signaling, leading to insulin-stimulated glucose uptake, and improved glycolysis, mitochondrial respiration, and fatty acid oxidation in myotubes (Tan et al., 2015). Inhibition of class I HDACs has been shown to be beneficial *in vivo*, in obese diabetic (db/db) mice, by improving insulin sensitivity and reducing body weight. Two different HDACi, i.e., SAHA and MS-275, promote PGC-1alpha-dependent mitochondriogenesis and oxidative metabolism in skeletal muscle, leading to higher energy expenditure in db/db mice (Galmozzi et al., 2013). Considering that MS-275 is a class I HDACi, unlike SAHA which is a pan-HDACi, it is likely that the protective effects on db/db mice were mediated by class I HDACs. Indeed, by knocking down the expression of each member of class I HDACs in muscle cells, Galmozzi et al. demonstrated that HDAC3 is the main driver of the metabolic rearrangement mediated by MS-275 in obese diabetic mice (Galmozzi et al., 2013). Importantly, the beneficial effects of the class I HDACi MS-275 of improved glucose tolerance and metabolic profile were also confirmed in other murine models of insulin resistance, such as HFD-induced obesity (Ferrari et al., 2016) or HFD/high fructose diet mice (Lee et al., 2020). Moreover, MS-275 protects myotubes from palmitate-induced lipotoxic conditions, by improving oxidative metabolism and by reducing the expression of inflammatory cytokines (Lee et al., 2020). In these experimental conditions, MS-275 effect was also mainly due to the inhibition of HDAC3 activity (Lee et al., 2020).

Importantly, despite their well-documented functions in muscle metabolism, the class II selective HDACi MC1568 did not recapitulate the MS-275 beneficial effects seen in obese diabetic mice (Galmozzi et al., 2013). However, pharmacological inhibition of class II HDAC deacetylase activity may not interfere with their main biological activity, which is believed to be more chaperone-like than deacetylase. Thus, the class II HDACi Scriptaid, which disrupts class IIa HDAC/Corepressor interactions, triggered MEF2-dependent metabolic adaptation in skeletal muscle, mimicking the exercise adaptive response (Gaur et al., 2016). In skeletal muscle, Scriptaid increased mitochondriogenesis and mitochondrial function, affecting whole-body energy expenditure and lipid oxidation. Systemically, Scriptaid administration also reduced blood lipids and glucose levels (Gaur et al., 2016). Moreover, Scriptaid treatment enhanced muscle insulin action and cardiac function in HFD-induced obesity in mice, by increasing fatty acid oxidation, energy expenditure, and lipid oxidation. However, Scriptaid administration did not ameliorate obese mice body weight or composition, likely because it increased mouse food intake (Gaur et al., 2016; Gaur et al., 2017).

Sirtuins are believed to be the major mediators of healthy metabolic responses to caloric restriction and exercise. This is true especially for SIRT1 and SIRT6, whose overexpression in mice recapitulate many of the physiological effects of caloric restriction and exercise (Bordone et al., 2007; Kanfi et al., 2012). Moreover, SIRT1 overexpression results in protection against diabetes and HFD-induced obesity in mice, ameliorating glucose tolerance and exerting anti-oxidative and anti-inflammatory properties (Banks et al., 2008; Pfluger et al., 2008). Of note, genetic polymorphisms on *Sirt1* and *Sirt2* genes have been associated with T2D development and insulin resistance (Maeda et al., 2011; Liu et al., 2018). Considering the positive effects on body metabolism when Sirtuins are overexpressed, the development of “Sirtuin activating compounds” (STACs) or the discovery of natural nutrients able to trigger sirtuin activation has been a major goal in the last 20 years (Howitz et al., 2003; Hubbard and Sinclair, 2014). As opposed to inhibitors, activating compounds are generally more specific, thereby presenting fewer side effects (Zorn and Wells, 2010).

Numerous natural phytochemicals with anti-diabetic activity (due to their effects on SIRT1 levels or activation) have been characterized, including quercetin (Oboh et al., 2016), fisetin (Liou et al., 2018), curcumin (Mohammadi et al., 2021), berberine (Yin et al., 2008), and sesamine (Majdalawieh et al., 2020; Kou et al., 2022). In addition, recent studies have unveiled the effectiveness against insulin resistance of new active components from plants, due to their ability to increase SIRT1 levels or activation, including Kaempferol3-O-rutinoside (KOR) from the leaf of *Antidesma acidum* (Kashyap et al., 2023), or the Jiangtang Sanhao formula, a mix of plants from traditional Chinese medicine (Ye et al., 2022), or the plant hormone Abscisic acid (ABA) (Spinelli et al., 2021). These discoveries have given rise to a novel category of food named Sirtfoods™, which have pleiotropic health functions by mitigating metabolic disorders (Akan et al., 2022). Sirtfoods™ comprises recipes for twenty kinds of fruits and vegetables supported by solid mechanistic and structural studies of the interactions between such functional foods and Sirtuins. However, the major limitation of nutritional approaches is the difficulty in monitoring the consumption of functional foods. Thus, being easier to monitor, the use of STACs is preferred in the fight of insulin resistance.

One of the most studied STACs is resveratrol, a natural phenol contained in skins of grapes and berries, able to activate SIRT1 by more than 10 folds (Howitz et al., 2003; Gertz et al., 2012). Following some preclinical evidence on its protective effects on metabolic diseases in rodents (Baur et al., 2006; Lagouge et al., 2006), resveratrol has been used in numerous clinical trials for the treatment of insulin resistance in metabolic syndromes and diabetes (Yoshino et al., 2012; Knop et al., 2013; Poulsen et al., 2013; Anton et al., 2014; Méndez-Del Villar et al., 2014; Kjær et al., 2017; Poulsen et al., 2018; Roggerio et al., 2018; Nocella et al., 2019; Walker et al., 2019). However, in most of these clinical trials resveratrol had no effects on patients and, when positive effects were registered, resveratrol concentrations were high (over 500 mg/day), implying that the bioavailability is a major obstacle for achieving therapeutic effects with this natural product.

Metformin is another promising medicine (FDA-approved in 1994) which elevates SIRT1 levels and activity (Tan et al., 2016). Thus far, over a thousand of clinical trials of metformin for the treatment of diabetes have been completed (ClinicalTrials.gov, 2022). In one of the largest randomized clinical trials including more than 3,000 American adults at high risk for the development of T2D, metformin treatment reduced the incidence of T2D by 31% compared to placebo, in a follow-up of 2.8 years (Iabetes et al., 2002). Of note, lifestyle interventions, consisting of a healthy low-calorie, low-fat diet in combination with physical activity of moderate intensity for at least 150 min per week, reduced the incidence of T2D by 58% compared to placebo (Iabetes et al., 2002), confirming the importance of combined approaches.

Several clinical trials for the treatment of diabetes have been completed or are currently ongoing involving numerous different STACs, such as SRT501, SRT2183, SRT1720, SRT1460, SRT1720, and SRT2379 (Song et al., 2018). Despite our growing knowledge regarding the bioavailability, toxicity, and administration dosage of the numerous STACs, no definitive treatment for diabetes has been approved. The need for developing more selective Sirtuins activators is still crucial. To this end, virtual screening has been used recently (Abbotto et al., 2022). Long-term rigorous studies, with validated outcomes compared to placebo groups are needed in order to confirm the effectiveness and the safety of these different STACs on diabetes.

The class IV member of HDACs, HDAC11, is emerging as an important metabolic hub for multiple tissues and organs (Bagchi et al., 2018; Sun et al., 2018). Moreover, HDAC11 is a key regulator of obesity, since the deletion of HDAC11 counteracts HFD-induced obesity, and reduces hepatic damage while increasing insulin sensitivity in mice (Bhaskara, 2018). Thus, selective and specific HDAC11 inhibitors should be available and tested as therapeutic molecules for the treatment of metabolic diseases.

Considering the specific functions of HDACs, we do not believe that developing pan-HDAC inhibitors is always a good strategy. Instead, it is important to define their specific function and finely tune the spatial-temporal parameters and specificity of the treatment. Such approach would improve the outcome of the therapeutic intervention and reduce the incidence of side effects. From the above studies, it seems that inhibiting class I HDACs or activating Sirtuins are two promising tools in the fight of insulin resistance. However, despite the vast preclinical evidence (Table 2), the US FDA approval of four HDACi (i.e., SAHA, Belinostat, Panobinostat, Romidepsin), (and one by China FDA (i.e., Chidamide)), and with more than twenty HDACi currently in clinical trials as anti-cancer drugs, there are at present no ongoing clinical trials involving the use of HDACi for the treatment of insulin resistance diseases.

**TABLE 2:** Potential therapeutic applications for the use of HDACi in metabolic diseases.

HDAC inhibitors	Selectivity	Experimental model	Disease	Outcome	References
Sodium butyrate	pan-HDACi	HFD mice Muscle cells	Obesity	**↑** insulin sensitivity	Gao et al. (2009); Chriett et al. (2017)
			Type 2 diabetes	**↑** energy expenditure	
SAHA	pan-HDACi	db/db mice HFD mice	Obesity	**↑** insulin sensitivity	Galmozzi et al. (2013); Kumar and Datta (2022)
			Type 2 diabetes	**↑**oxidative metabolism	
MS-275	class I HDACi	Myotubes db/db mice HFD mice HFD/high fructose diet mice	Obesity	**↑** insulin sensitivity	Galmozzi et al. (2013); Ferrari et al. (2016); Lee et al. (2020)
			Type 2 diabetes	**↑** oxidative metabolism	
				**↓** inflammation	
				**↑**white fat browning	
Scriptaid	Disrupts class IIa HDACs/Corepressor complex	HFD mice	Obesity	**↑**energy expenditure	Gaur et al. (2017)
				**↑**lipid oxidation	
				**↓** triglycerides	

## 7 Conclusion

Skeletal muscle metabolism is finely regulated by HDACs, thus rendering these enzymes a promising therapeutic target in pathophysiological conditions (Table 2). Although the specific functions of HDACs in skeletal muscle are still under investigation, several HDACi or Sirtuin activators have been proposed as a potential pharmacological approach for the treatment of insulin resistance, obesity, and type 2 diabetes. However, most of the considered treatments are broad-spectrum, such as class I HDACi or STACs. This non-selective strategy could be improved by a better understanding of the functions of HDACs in specific cell types, or spatiotemporal districts in skeletal muscle, which will likely increase the drug’s effectiveness in the treatment of metabolic diseases.
