# Corrigendum: Histone deacetylase functions and therapeutic implications for adult skeletal muscle metabolism

**DOI:** 10.3389/fmolb.2023.1201886

**Published:** 2023-04-20

**Authors:** Susanna Molinari, Carol Imbriano, Viviana Moresi, Alessandra Renzini, Silvia Belluti, Biliana Lozanoska-Ochser, Giuseppe Gigli, Alessia Cedola

**Affiliations:** ^1^ Department of Life Sciences, University of Modena and Reggio Emilia, Modena, Italy; ^2^ Institute of Nanotechnology, Department of Physics, National Research Council (CNR-NANOTEC), Sapienza University of Rome, Rome, Italy; ^3^ DAHFMO Unit of Histology and Medical Embryology, Sapienza University of Rome, Rome, Italy; ^4^ Department of Medicine and Surgery, LUM University, Casamassima, Italy; ^5^ Institute of Nanotechnology, National Research Council (CNR-NANOTEC), Lecce, Italy

**Keywords:** HDACs, sarcopenia, type 2 diabetes, neurogenic muscle atrophy, sirtuins, glucose uptake, fatty acid oxidation, metabolic flexibility

In the published article, there was an error in affiliation(s) of Silvia Belluti and Alessia Cedola. Instead of Silvia Belluti^3^ and Alessia Cedola^1^, it should be “Silvia Belluti^1^ and Alessia Cedola^2^.”

The authors apologize for this error and state that this does not change the scientific conclusions of the article in any way. The original article has been updated.

